# The Takeda Three Colors Combination Test: A Screening Test for Detection of Very Mild Alzheimer's Disease

**DOI:** 10.1155/2014/907316

**Published:** 2014-10-19

**Authors:** Shinya Takeda, Kayo Tajime, Toshiatsu Taniguchi

**Affiliations:** ^1^Department of Clinical Psychology, Tottori University Graduate School of Medical Sciences, 86 Nishicho, Yonago, Tottori Prefecture 683-8503, Japan; ^2^Department of Psychiatry, Tottori Seikyo Hospital, 458 Suehiro-onsen, Tottori, Tottori Prefecture 680-0833, Japan

## Abstract

*Background*. Alzheimer's disease (AD) is the most common type of dementia and is prevalent worldwide. It is expected that AD, for which aging is a risk factor, will increase in the future. Because early detection of AD has become increasingly important, promoting demand for screening tests with adequate sensitivity. In this study, we examined the usefulness of the Takeda Three Colors Combination Test (TTCC) for screening of the very mild AD and amnestic mild cognitive impairment (aMCI). *Methods*. 154 senior persons participated in the research: 55 with very mild AD, 45 with aMCI, and 54 control group. The TTCC, which was a colored cards configuration memory task, was examined for sensitivity and specificity. *Results*. The sensitivity of the TTCC was 76% and 47% for the very mild AD and aMCI groups, and the specificity was 83%. Conducting TTCC (including instruction and evaluation) was accomplished within 2 minutes for all subjects. *Conclusion*. The TTCC is useful screening test for early detection of AD. Furthermore, administration time is short and requires no special training or skills. Thus, we believe the TTCC shows great potential for use as an AD screening test by a general practitioner in communities worldwide.

## 1. Introduction

Alzheimer's disease (AD) is the most common type of dementia and is prevalent worldwide [1, 2]. The risk factor of AD is aging [3]. With an increase of the elderly population as reported by the United Nations [4], it is expected that the AD population will only grow in the future. Although treatment for AD has yet to be established, medications such as acetylcholinesterase inhibitors can delay the progress or improve symptoms of AD in its early stages and are readily available. With a projected increase in the AD population and rising awareness of early intervention measures, early detection has become an increasingly important issue, generating demand for screening tests with higher sensitivity.

AD dysfunction is not readily identifiable in the early stage and is difficult to recognize by patients themselves or their families and so early consultation with a medical specialist is all too rare. Moreover, a majority of the world's elderly live in developing countries, a trend which is likely to continue [5], meaning that the greater part of AD patients will be living in developing countries [6] where there exists a lack of specialists in dementia. Given this global situation, it is necessary to come up with a means to conduct early detection of AD by a general practitioner (GP), someone in the community to whom patients have easy access. At present, the rate of general screening tests conducted by GPs is reported to be low [7] as too much time and advance training are required. What is needed then is a simple screening test with high sensitivity: one that a busy GP can use for early detection of AD.

Most screening tests up to now include questions which measure the patient's language skills, often assuming the patient has had formal education [8]. There are people in many parts of the world, however, who are illiterate or lack years of formal education. Also, considering the elderly can be easily fatigued, screening tests must be brief and the content motivating for the examinee. It is suggested that an AD screening test for use internationally (a) facilitates early detection, (b) requires no special training to administer, (c) poses no burden or strain on the examinee, (d) is accomplished in a short time, (e) is easy to evaluate, and (f) is neutral in terms of the examinee's literacy, education, and culture.

With these conditions in mind, Takeda et al. [9] have developed the Takeda Three Colors Combination Test (TTCC), an easier and quicker screening test for dementia. The TTCC task requires the examinee to reconstruct a figure in a procedure described as follows. First, the examinee is shown a figure composed of three square, colored cards (red, blue, and yellow) for five seconds. The figure is hidden. The examinee then performs an interference task (the reverse-backward testing). The examinee then arranges three cards to match the model shown previously. If the examinee makes the same figure as the model, he or she is regarded as normal. If he or she is unable to make the same figure, AD is suspected. It also demonstrates high discriminative power for mild AD with a sensitivity of 85% and specificity of 87%. In regard to reliability, the retest showed a high value in both concordance rate (88%) and correlation coefficient (*φ* = 0.76).

However, Takeda et al. [9] had never conducted a study focusing on very mild AD, that is, the early stage of AD. To detect AD early, a powerful screening test for very mild AD is needed. On the other hand, the previous studies by Takeda et al. [9] did not discuss sensitivity of the TTCC for mild cognitive impairment (MCI). MCI is classified into several types, and amnestic MCI (aMCI) is thought to lead to AD. To prevent AD and implement early intervention, screening tests are required to selectively detect aMCI. Therefore, the present study was designed to assess the efficacy of the TTCC for early detection of AD. Patients with either very mild AD or aMCI were enrolled in the study. The symptoms of very mild AD and aMCI are often similar; thus, we also discuss whether very mild AD and aMCI can be detected using reverse-backward presentation of figures (an interference task of TTCC).

## 2. Methods and Materials

### 2.1. Subjects

The subjects were 154 persons (men and women) divided into three groups: very mild AD, aMCI, and control group (Table 1). The inclusion criterion was being aged 60 years or over. Exclusion criteria were psychiatric diseases, delirium, verbal incomprehension including aphasia, and neurological disease. The people in the very mild AD group (*n* = 55) met the probable AD criteria of National Institute of Neurological and Communicative Disorders and Stroke and the Alzheimer's Disease and Related Disorders Association (NINCDS-ADRDA) [10], scoring more than 21 on a Mini-Mental State Examination (MMSE) [11], and received a Clinical Dementia Rating (CDR) [12] of 0.5 in all 6 areas. The people in the aMCI group (*n* = 45) met the Petersen's criteria [13], scoring more than 24 on MMSE, and received a CDR of 0.5 in “memory” and a CDR of 0 in other areas. The control group (*n* = 54) comprised people who had no complaint of memory lapse, were not diagnosed as dementia, scored 28 or greater on a MMSE, and scored 0 on a CDR. We explained the object and methods of the research to the subjects (or their families) and obtained their consent in advance of their participation. The Ethics Board of Tottori University School of Medical Sciences approved all procedures.

Comparing the average value of age in 1-way ANOVA, the control group was significantly younger than other groups (*F* = 20.6, *P* ≤ 0.001). There were no significant differences in gender (*χ* ^2^ = 0.13, n.s.) among groups using the Chi square test (group [3]  × gender [2]). Comparing the average value of MMSE scores for 1-way ANOVA, the control group was significantly higher than other groups, and the aMCI group was significantly higher than very mild AD group (*F* = 144.6, *P* ≤ 0.001).

### 2.2. The TTCC Featured the Following

*Procedures*

**Figure 1:**
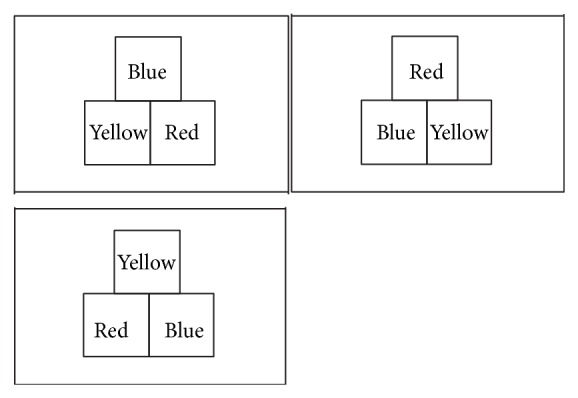
The arrangement of three colored squares that should be remembered and reproduced later after an interference task using three wooden colored cards. One of 3 models was shown to the examinee.

1. Show examinee three wooden colored cards (red, blue, and yellow, each five centimeters square and five millimeters thick) and confirm that the examinee can distinguish the colors.
2. Explain to the examinee as follows: “I'll show you a figure for five seconds, please remember it. I'll ask you to make the same figure from memory later using these three cards. Do you understand?” If the examinee understands, hide the cards and proceed to the next step. If the examinee does not understand, explain again.
3. Present the card showing the three colored squares in a certain arrangement (model: one of 3 models was shown to the examinee; Figure 1) for five seconds and then hide it quickly. Ask the examinee to repeat the sets of numbers immediately in reverse sequence (“4-1-7”, “5-2-4-9”).
4. After the examinee has done this, stack the three cards and hand them to the examinee. Ask the examinee “Please make the same figure I showed you earlier.” Allow one minute to complete the task.

*Conditions for Stopping the Test.* Stop the test under either of the following conditions.

1. The examinee is not able to complete the arrangement within one minute following instruction.
2. The examinee completes the arrangement within one minute following instruction.

*Evaluation.* If the examinee succeeded in making exactly the same figure as the model, he or she is evaluated as being normal. If the arrangement of the color cards differs from the model, AD is suspected. Evaluation does not include accuracy of the recitation in reverse of number sets.

Any of the following cases is also regarded as incorrect.

1. The cards are overlapped.
2. The top square is placed off-center, such that the ratio of the lengths of the bottom side contacting the two lower squares exceeds 1.5.
3. Cards are arranged more than five millimeters apart.

### 2.3. Statistical Analysis

The TTCC was conducted on all examinees, in the following order: TTCC, MMSE, and CDR. The examiner was not told which group the examinees belonged to. With TTCC, we allotted “1” to a correct response and “0” to an incorrect response as dummy variables. Because a significant difference for age was obtained in each group, the differences among the very mild AD, aMCI, and control group were examined by logistic regression analysis to adjust for age factor using “TTCC results” as a dependent variable and “group” and “age” as independent variables. The difference of the reverse-backward testing between the very mild AD group and aMCI group was examined by a Chi square test.

## 3. Results

For the very mild AD group, 13 responded correctly and 42 responded incorrectly to the TTCC. For the aMCI group, 24 responded correctly and 21 responded incorrectly to the TTCC. For the control group, 45 responded correctly and 9 responded incorrectly to the TTCC (Table 2). The sensitivity of the TTCC was 76% and 47% for the very mild AD and aMCI groups, and the specificity was 83%. As the result of logistic regression analysis, group was the only significant item. The odds ratio of incorrect response to TTCC was 16.2 (95% confidence interval = 6.3–41.7, *P* ≤ 0.001) for very mild AD compared with control and was 4.4 (95% confidence interval = 1.7–11.0, *P* ≤ 0.05) for aMCI compared with control.

For the very mild AD group, 47 responded correctly and 8 responded incorrectly to the reverse-backward testing with 3 figures. For the aMCI group, 43 responded correctly and 2 responded incorrectly to the reverse-backward testing with 3 figures. On the other hand, for the very mild AD group, 22 responded correctly and 33 responded incorrectly to the reverse-backward testing with 4 figures. For the aMCI group, 19 responded correctly and 26 responded incorrectly to the reverse-backward testing with 4 figures. There were no significant differences in the reverse-backward testing (3 figures: *χ* ^2^ = 2.8; 4 figures: *χ* ^2^ = 0.05, n.s.) between groups using the Chi square test.

Conducting TTCC (including instruction and evaluation) was accomplished within 2 minutes for all subjects. No refusal or resistance to this test was observed among any of the subjects.

## 4. Discussion

In the present study, we examined the usefulness of TTCC for screening of very mild AD and aMCI. The results of logistic regression analysis show that patients with very mild AD tend to respond incorrectly to TTCC 16 times as often as examinees evaluated as normal when the rate of their incorrect response is 1. As for the power of the TTCC to detect very mild AD, sensitivity was 76%, and the specificity was 83%. The lower sensitivity compared with results of an earlier study [9] is likely due to the fact that subjects in the present study were very mild AD compared with the mild AD examinees of the former study. However, the sensitivity value of the present study can be compared favorably with those of other screening tests [14–16] from previous research where many of the subjects had moderate to severe dementia, conceivably giving those tests high sensitivity values. Because the present study did not involve moderate and severe patients, the sensitivity is considered satisfactory. Takeda et al. [9] claim that the relatively high sensitivity for AD shown in the screening is explained by TTCC's fidelity in detecting impairment in recent memory and space perception, both of which are seen at the early stage of AD [17, 18]. They suggest that sufficient screening tests can be made with fewer test items if those items test for cognitive dysfunctions which appear in the early stage of AD.

MMSE, used widely to screen for dementia, shows poor sensitivity to the early stage or when the condition is mild [19], a limitation which can be seen when sensitivity falls to as much as 54% [20] for dementia groups scoring more than 20. Since the subjects in the very mild AD group of the present study scored more than 21 in MMSE, with some over the cutoff point, TTCC's sensitivity to very mild AD is considered to be quite high.

The results of logistic regression analysis suggest that patients with aMCI tend to respond incorrectly to TTCC 4.4 times as often as examinees evaluated as normal when the rate of their incorrect response is 1. However, we found that sensitivity of the TTCC test for aMCI was only 47%. This result indicates that the TTCC test is not a reliable tool for aMCI screening. However, the percentage of patients who recover from aMCI within 2–5 years is approximately 40% [21, 22]; accordingly, we believe that recovered patients should be excluded from the screening tests to selectively detect aMCI that is likely to progress to AD. Deterioration in recent memory and visual-spatial cognitive functions is considered a risk factor for progression from MCI to AD [18]. According to this notion, the TTCC test may be useful for detection of possible aMCI because this test evaluates recent memory and visual-spatial cognitive functions. More research is required to determine whether TTCC test results can be used to detect aMCI that is likely to progress to AD.

When we compared the results of the interference task between the very mild AD and the aMCI groups, no significant differences were found in reverse-backward testing with 3 and 4 figures. This finding indicates that it is difficult to distinguish very mild AD and aMCI using the interference task of TTCC. The reverse-backward tests assess attention function; therefore, the results of this study show that there are no remarkable differences in attention function between patients with very mild AD and those with aMCI.

Screening tests for dementia used worldwide include the Rapid Dementia Screening Test (RDST), the Memory Impairment Screen (MIS), and the Montreal Cognitive Assessment (MoCA), which are simple, convenient, and reliable methods, with high sensitivity [14, 15, 23]. There are four differences between TTCC and these tests. (1) TTCC can detect patients' deteriorated functions, which are initial symptoms of very mild AD. Patients with AD often develop deterioration in recent memory and visual-spatial cognitive functions at an early stage [17, 18]. However, except for TTCC, none of the existing tests contain an evaluation item to assess the deterioration in both recent memory and visual-spatial cognitive functions. (2) TTCC consists of only one task. To screen people for dementia, the conventional methods require examinees to perform several tasks. Elderly people get tired easily; therefore, to encourage them to undergo an examination, it is preferable that the screening test consists of the smallest possible number of tasks. Therefore, TTCC can be useful because it requires only one task. (3) The duration of TTCC examination is comparatively short. Patients had to traditionally spend 4–15 min to complete all screening procedures from beginning till evaluation; however, TTCC can be finished within 2 min. According to a previous study [24], the average duration of consultation in Europe is 10.7 min. TTCC can be the most convenient testing method for doctors who need to diagnose dementia as early as possible. (4) TTCC is easily to perform. There are no data on conventional screening tests that investigate the percentage of examinees who drop out in the middle of a test. Our study demonstrated that all patients completed the TTCC task without any refusal. This finding suggests that TTCC is a stress-free screening method.

In conclusion then the TTCC is useful screening test for early detection of AD. The content of the test itself is extremely simple and unbiased in regard to examinee literacy. The equipment is cheap. Administration time is short and requires no special training or skills. And Takeda and Tajime [25] revealed that educational attainment of the persons did not affect the performance of the TTCC. For these reasons, we believe the TTCC shows great potential for use as an AD screening test by GP in communities worldwide.

Finally, we must discuss questions that remain to be answered. To begin with, it is noted that TTCC is a simplified screening test and as such cannot be used on its own to diagnose AD. Tasks for the present study were structured to be free from literacy and cultural affects, with the aim of making TTCC suitable for use in developing countries where a large number of senior people and patients with AD are found. We have yet to administer tests in these regions, however. In order to study its effectiveness, TTCC must be administered in countries worldwide, including developing countries.

## 5. Conclusions

The TTCC is useful screening test for early detection of AD. Furthermore, administration time is short and requires no special training or skills. Thus, we believe the TTCC shows great potential for use as an AD screening test by a general practitioner in communities worldwide.

## Figures and Tables

**Table 1:** Demographic data.

Classification	Number	Age years	Sex (male : female)	MMSE
Very mild AD	55	76.7 ± 5.9	20 : 35	23.1 ± 2.6
aMCI	45	77.4 ± 4.2	16 : 29	26.8 ± 1.8
Control	54	71.2 ± 5.7	21 : 33	29.2 ± 0.8

**Table 2:** The results of the TTCC and the reverse-backward testing.

Group	TTCC		Backward 3-digit span		Backward 4-digit span	
	Correct response	Incorrect response	Correct response	Incorrect response	Correct response	Incorrect response
Very mild AD	13	42	47	8	22	33
aMCI	24	21	43	2	19	26
Control	45	9	49	5	30	24
